# Increase in Congenital Toxoplasmosis During the COVID-19 Pandemic in the US

**DOI:** 10.3390/pathogens13110937

**Published:** 2024-10-28

**Authors:** Despina G. Contopoulos-Ioannidis, Valerie Bonetti, Jose G. Montoya

**Affiliations:** Dr. Jack S. Remington Laboratory for Specialty Diagnostics, Palo Alto Medical Foundation, Sutter Health, Palo Alto, CA 94301, USA; valerie.bonetti@sutterhealth.org (V.B.); jose.montoya2@sutterhealth.org (J.G.M.)

**Keywords:** congenital toxoplasmosis, acute toxoplasmosis during pregnancy, COVID-19

## Abstract

During the COVID-19 pandemic, prenatal care services were disrupted. We analyzed the trajectory slopes of cases of congenital toxoplasmosis (CT) and acute toxoplasmosis during pregnancy from 2019 to 2022 (to reflect the cases during the pandemic years 2020–2022) vs. 2000 to 2019, using data from the Remington Laboratory, the National Reference Center for Toxoplasmosis in the US. During the pandemic, there was a statistically significant upward trajectory in the yearly number of CT cases. Monitoring of this trend is needed.

## 1. Introduction

A rise in diverse opportunistic infections has been described during the pandemic. Also, during the COVID-19 pandemic prenatal care services were disrupted and pregnant women often feared going to medical facilities for their prenatal care. We analyzed the trajectory slopes of cases of congenital toxoplasmosis (CT) and acute toxoplasmosis during pregnancy (ATP) during the COVID-19 pandemic period compared to a pre-pandemic period (2000–2019).

## 2. Methods

In this retrospective cohort study, we analyzed data from the Remington Laboratory, the National Reference Laboratory for Toxoplasmosis in the US. We extracted information on the number/ per year of CT cases in infants < 12 months and of ATP cases. CT cases were identified based on a specific code routinely used at the Remington lab that identifies infants born to mothers with acute *Toxoplasma* infection during pregnancy who have a positive neonatal IgG Dye test and a positive IgM ISAGA or IgA Elisa, and/or a positive *T. gondii* PCR in amniotic fluid or in any of the infant’s body fluids (e.g., cerebrospinal fluid, blood, urine) and for which anti-*Toxoplasma* therapy was recommended by the medical consultant of the lab. Cases of ATP were identified based on a specific code routinely used at the Remington lab for pregnant women who have a serologic profile of acute *Toxoplasma* infection and for which anti-*Toxoplasma* therapy was also recommended by the medical consultant of the lab. The serologic criteria for acute *Toxoplasma* infection routinely used at the Remington lab include the following two composite criteria: (a) IgG Dye test titer ≥ 1:1024 PLUS IgM ELISA ≥ 5.0 PLUS acute pattern in the differential agglutination AC/HS test PLUS low IgG avidity, PLUS in some cases also of IgA ELISA ≥ 2.1 or IgE ELISA ≥ 1.9; or (b) IgG Dye test titer ≤ 1:512 PLUS IgM ELISA ≥ 5.0 PLUS acute pattern in the differential agglutination AC/HS test PLUS either IgA ELISA ≥ 2.1 or low IgG avidity (<10). Pregnant women meeting any of the above two composite criteria are likely to be infected within less than 6 months from the time of testing.

We also calculated the total number of infants < 12 months of age and pregnant women tested for toxoplasmosis in Remington lab with a *Toxoplasma* IgG Dye test during this period. We calculated the yearly number of cases and yearly incidence rate (IR) thereof of CT and ATP (IR-CT: number of CT/100 tested infants < 12 months and IR-ATP: number of ATP/100 tested pregnant women, respectively). We calculated the trajectory slopes (and 95% confidence intervals thereof) of the number of cases and IR thereof of CT and ATP for the period 2019–2022 ( to reflect the cases during the pandemic years 2020–2022) vs. 2000–2019, using ARIMA time-series models (p,d,q). We also estimated the change in the trajectory slope between the two periods. *p*-values < 0.05 were considered significant.

## 3. Results

In the 20-year pre-pandemic period (2000–2019) there were 196 CT cases among 8194 tested infants < 12 months and 814 ATP cases among 33,345 tested pregnant women. The respective numbers during 2020–2022 were 43 CT cases among 1833 tested infants < 12 months and 65 ATP cases among 1983 pregnant women. In 2022, there was the highest number of CT cases (21 in 2022 vs. 6 in 2019). A similarly high number of ATP cases was also seen in 2022 (22 in 2022 vs. 16 in 2019).

### 3.1. Number of Cases

**Figure 1:**
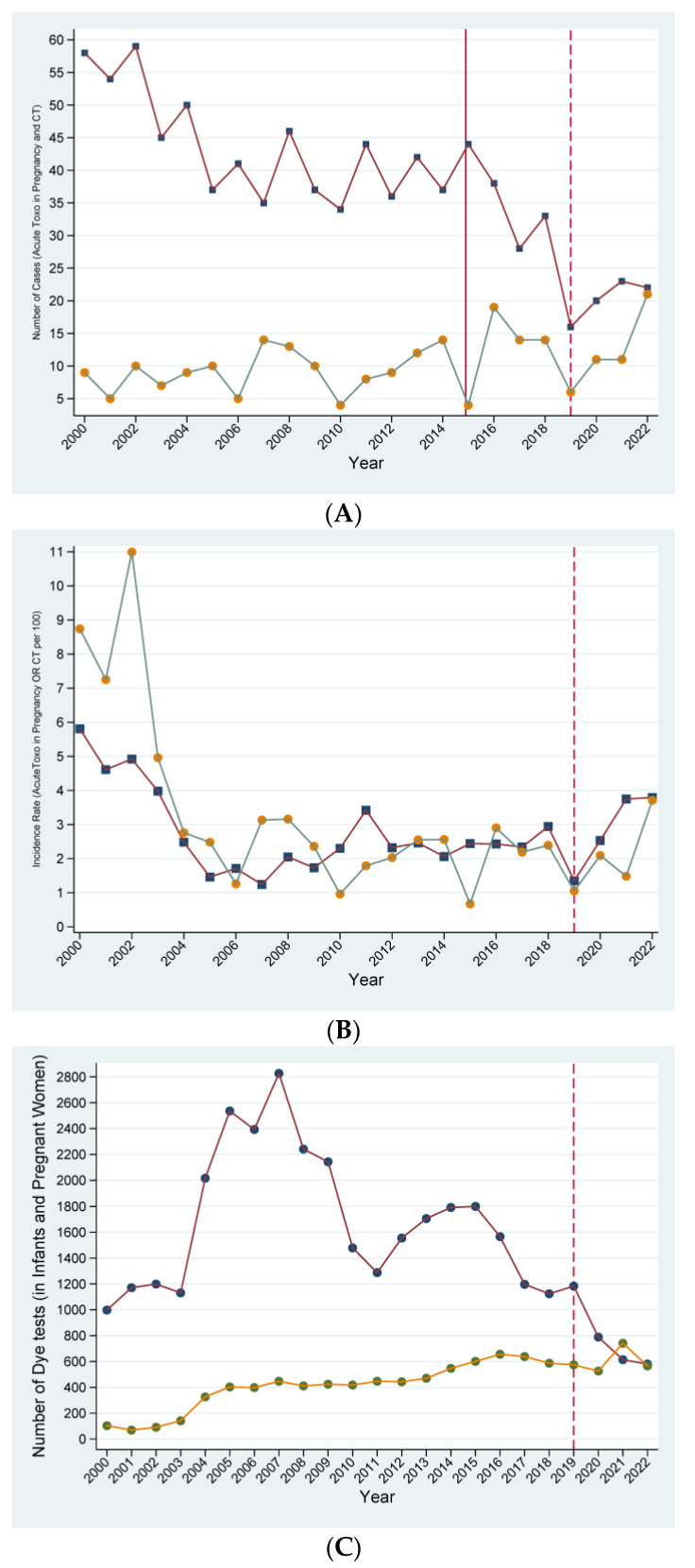
(**A**): Number of cases of congenital toxoplasmosis (CT) and acute toxoplasmosis during pregnancy (ATP) (2000–2022); (**B**) incidence rate of CT and ATP (cases/100 tested) (2000–2022); (**C**) Number of tested infants < 12 months of age and number of tested pregnant women (2000–2022) (Footnote: orange circles/green lines: infants < 12 months of age, blue squares/red lines: pregnant women).

From 2019 to 2022 there was a significant upward trajectory slope in the number of CT cases (trajectory slope: +5 CT cases/year (95%CI: +0.38 to +9.62; *p* = 0.034). In the preceding period 2000–2019 there was a non-statistically significant trend in the trajectory slope in the yearly number of CT cases (trajectory slope: +0.69 CT cases/year (95%CI: −1.72 to +1.86; *p* = 0.940) (Figure 1A, Table 1). The change in the trajectory slope of CT between the two periods (pandemic vs. pre-pandemic) was +1.71 CT cases/year (*p* = 0.088).

From 2019 to 2022 there was a non-statistically significant upward trend in the trajectory slope of the number of ATP cases (trajectory slope: +2 ATP cases/year (95%CI: −2.53 to +6.54; *p* = 0.39). In the preceding period, 2000–2019, there was a significant downward trajectory (trajectory slope: −1.83 ATP cases/year (95%CI: −3.44 to −0.22; *p* = 0.026) (Figure 1A). The change in the trajectory slope in ATP cases between the two periods (pandemic vs. pre-pandemic) was +1.56 (*p* = 0.119).

### 3.2. Incidence Rates

The trajectories for the IR-CT and IR-ATP for 2019–2022 showed a non-statistically significant change in the number of CT cases/ per 100 tested infants per year and a non-statistically significant change in the number of ATP cases/ per 100 tested pregnant women per year (Figure 1B, Table 1). The trajectory slope for IR-CT was +0.89 (95%CI: −0.48 to +2.26; *p* = 0.205), and the trajectory slope for IR-ATP was +0.81 (95%CI: −6.11 to +7.73; *p* = 0.818) (Figure 1B, Table 1). The trajectory slope for the IR-CT for the preceding period 2000–2019 was −0.38 (95%CI: −1.06 to + 0.30; *p* = 0.274), and for the IR-ATP was −0.22 (95%CI: −0.54 to +0.10; *p* = 0.177) (Figure 1B). The change in the trajectory slopes in the IR-CT and IR-ATP between the two periods (2019–2022 vs. 2000–2019) was +1.63 (*p* = 0.104) for the IR-CT and +0.29 (*p* = 0.77) for the IR-ATP.

More specifically, the yearly IR-CT increased from 1.05 cases/100 tested infants (6/574) in 2019 to 3.71 cases/100 tested infants (21/566) in 2022, while there was no statistically significant change in the number of tested infants < 12 months from 2019 to 2022 (Figure 1C). The trajectory slope for the number of tested infants < 12 months per year had a non-statistically significant trend (trajectory slope: −2.67 [95%CI: −284.74 to +219.41; *p* = 0.981). The yearly IR of ATP increased from 1.35 cases/100 tested pregnant women (16/1183) in 2019 to 3.79 cases/100 tested pregnant women (22/580) in 2022, while the number of tested pregnant women had decreased from 2019 to 2022. There was a statistically significant negative trajectory slope for the number of tested pregnant women per year (trajectory slope: −201 [95%CI: −381.00 to −20.99; *p* = 0.029) (Figure 1C).

## 4. Discussion

During the pandemic, there was a significant upward trajectory in the number of CT cases per year diagnosed in Remington Laboratory, the National Reference Laboratory for toxoplasmosis in the US, with a peak of 22 CT cases noted in 2022, while there was non-statistically significant change in the yearly number of CT cases in the preceding 20 years. During the same period, an upward trend was also detected in the trajectory of the number of ATP cases per year, despite a preceding statistically significant downward trajectory in the number of ATP cases/per year in the 2000–2019 period. This increase occurred despite an overall decrease in the number of samples tested in Remington Laboratory ≥ 2020 due to programmatic changes in one major laboratory client and an associated reduction in the total number of tested pregnant women. Otherwise, there was no significant change in the number of tested infants < 12 months during the same period.

The etiology of this phenomenon remains unclear. Monitoring of this trend is necessary. Some possible hypotheses are that during the lockdowns, there was an increase in the contact of pregnant women with cats, a reduction of routine checks during pregnancy, and limited preventive guidance for pregnant women.

Additionally, it remains unclear whether, during the COVID-19 pandemic, co-infection of pregnant women with SARS-CoV2 (symptomatic or asymptomatic) and acute *T. gondii* infection could have increased the risk of vertical transmission of *T. gondii* to the fetus. Moreover, although cats have been documented to be infected with SARS-CoV2 [1,2,3,4] it remains unclear whether cats infected with SARS-CoV2 could also have increased shedding of *T. gondii,* after an acute *T. gondii* infection.

Several cases of opportunistic infections have been reported in COVID-19-infected patients [5,6]. Latent *Toxoplasma* infection may reactivate during COVID-19 infection and may remain undiagnosed due to overlapping symptomatology and lack of guidelines for routine screening for opportunistic parasitic infections during COVID-19 treatment [5,7]. This increase in opportunistic infections is likely to be due to the COVID-19 disease pathology and/or the extensive use of corticosteroids for the treatment of COVID-19 disease [7]. A case of CT after severe maternal COVID-19 infection has also been described [8]. Pregnant women with chronic latent *T. gondii* infection, in general, are not at risk for transmitting the infection to their fetus unless severely immunocompromised. It remains unclear whether pregnant women with chronic *Toxoplasma* infection could reactivate their latent *Toxoplasma* infection in the context of an acute SARS-CoV2 co-infection.

Some study limitations should be acknowledged. First, during the pandemic, we had data on CT and ATP for only 3 years; thus, the observed changes in the trajectories in the number of cases of CT and ATP should be viewed with caution. Second, we did not have information for social and other risk factor to further test possible explanations for this observation. Furthermore, we did not have information on the SARS-CoV2 status of the mothers or the babies with CT.

In conclusion, this report emphasizes the need for monitoring during the COVID-19 pandemic of CT and ATP cases. Given the widespread SARS-CoV2 infections among pregnant women, there is a need for systematic prenatal screening for toxoplasmosis in pregnant women. This report also probes important hypotheses that need further testing in future research, including the possibility of (a) increased risk for acquiring acute *Toxoplasma* infection among pregnant women who have close contact with cats, due to the possibility of increased *T. gondii* shedding in cats co-infected with SARS-CoV2 and *T. gondii*; (b) increased risk for vertical transmission of *T. gondii* infection in pregnant women with SARS-CoV2 and acute *Toxoplasma* coinfections and (c) increased risk for vertical transmission of *T. gondii* infection also in chronically infected pregnant women who acquire acute SARS-CoV2 infection during pregnancy, due to possible reactivation of latent toxoplasmosis in the context of acute COVID-19 disease.

## Figures and Tables

**Table 1:** Trajectory slopes and change in trajectory slopes between 2019–2022 * and 2000–2019.

	2019–2022 *	2000–2019	Change in Trajectory Slopes (2019–2022 vs. 2000–2019)
	Trajectory Slopes (by ARIMA time-series model) **	Trajectory Slopes (by ARIMA time-series model) ^¥^	
Congenital toxoplasmosis (N of cases/year)	+5; 95%CI: +0.38 to +9.62; (*p* = 0.034)	+0.69; 95%CI: −1.72 to +1.86 (*p* = 0.940)	+1.71 (*p* = 0.088)
Acute toxoplasmosis during pregnancy (N of cases/year)	+2; 95%CI: −2.53 to +6.54 (*p* = 0.39)	−1.83; 95%CI: −3.44 to −0.22 (*p* = 0.026)	+1.56 (*p* = 0.119)
Congenital toxoplasmosis (IR-CT cases/100 tested infants per year)	+0.89; 95%CI: −0.48 to +2.26 (*p* = 0.205)	−0.38; 95%CI: −1.06 to +0.30 (*p* = 0.274)	+1.63 (*p* = 0.104)
Acute toxoplasmosis during pregnancy (IR-ATP cases/100 tested pregnant women per year)	+0.81; 95%CI: −6.11 to +7.73 (*p* = 0.818)	−0.22; 95%CI: −0.54 to +0.10 (*p* = 0.177)	+0.29 (*p* = 0.77)

* The trajectory slopes from 2019 to 2022 reflect the cases during the pandemic years 2020–2022. ^¥^ For the period 2000–2019, an ARIMA model (1,1,0) was used for CT and ATP (number of cases and incidence rates). ** For the period 2019–2022, an ARIMA model (0,1,0) was used for CT and ATP (number of cases and incidence rates). Of note, for 2000–2019, the trajectory slope for the number of IgG Dye tests in infants < 12 months (by ARIMA (1,1,0)) was +22.38; 95%CI: −19.05 to +63.80; *p* = 0.290; and for 2019–2022 (by ARIMA (0,1,0)) was −2.67; 95%CI: −284.74 to +219.41; *p* = 0.981. For 2000–2019, the trajectory slope for the number of *Toxoplasma* IgG Dye tests in pregnant women (by ARIMA (1,1,0)) was +11.68; 95%CI: −182.60 to +205.97; *p* = 0.906 and for 2019–2022 (by ARIMA (0,1,0)) was −201; 95%CI: −381.00 to −20.99; *p* = 0.029.

## Data Availability

All data are reported in the paper.
